# Biocatalytic reductive amination as a route to isotopically labelled amino acids suitable for analysis of large proteins by NMR†

**DOI:** 10.1039/d3sc01718d

**Published:** 2023-10-26

**Authors:** Jack S. Rowbotham, Jake H. Nicholson, Miguel A. Ramirez, Kouji Urata, Peter M. T. Todd, Gogulan Karunanithy, Lars Lauterbach, Holly A. Reeve, Andrew J. Baldwin, Kylie A. Vincent

**Affiliations:** a Department of Chemistry, University of Oxford, Inorganic Chemistry Laboratory South Parks Road Oxford UK jack.rowbotham@manchester.ac.uk kylie.vincent@chem.ox.ac.uk; b Department of Chemistry, University of Oxford, Physical and Theoretical Chemistry Laboratory Oxford UK; c Technische Universität Berlin, Institute for Chemistry Straße des 17. Juni 135 10437 Berlin Germany; d Kavli Institute for Nanoscience Discovery, University of Oxford Oxford OX1 3QU UK andrew.baldwin@chem.ox.ac.uk

## Abstract

We demonstrate an atom-efficient and easy to use H_2_-driven biocatalytic platform for the enantioselective incorporation of ^2^H-atoms into amino acids. By combining the biocatalytic deuteration catalyst with amino acid dehydrogenase enzymes capable of reductive amination, we synthesised a library of multiply isotopically labelled amino acids from low-cost isotopic precursors, such as ^2^H_2_O and ^15^NH_4_^+^. The chosen approach avoids the use of pre-labeled ^2^H-reducing agents, and therefore vastly simplifies product cleanup. Notably, this strategy enables ^2^H, ^15^N, and an asymmetric centre to be introduced at a molecular site in a single step, with full selectivity, under benign conditions, and with near 100% atom economy. The method facilitates the preparation of amino acid isotopologues on a half-gram scale. These amino acids have wide applicability in the analytical life sciences, and in particular for NMR spectroscopic analysis of proteins. To demonstrate the benefits of the approach for enabling the workflow of protein NMR chemists, we prepared l-[α-^2^H,^15^N, β-^13^C]-alanine and integrated it into a large (>400 kDa) heat-shock protein oligomer, which was subsequently analysable by methyl-TROSY techniques, revealing new structural information.

## Abstract 2

We demonstrate an atom-efficient and easy to use H_2_-driven biocatalytic platform for the enantioselective synthesis of isotopically labelled amino acids for protein NMR studies.

## Graphical abstract

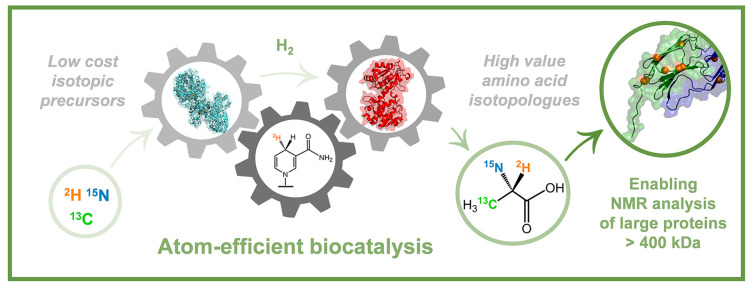

## Introduction

Selective isotopic labelling is widely used for probing chemical and biochemical systems.^1,2^ Deuterium (^2^H) is a particularly attractive substitution as the heavy isotope of hydrogen can be installed into a wide range of molecular sites to facilitate analysis by a suite of techniques.^3^ Notably, the development of isotopically labelled amino acids has been transformative for the analysis of proteins, enabling atomic resolution mechanistic, dynamical, and structural studies *via* a range of experimental techniques.^3–7^ Solution NMR spectroscopy particularly benefits selective isotopic enrichment, where the combination of carefully matched labelling schemes and experiments can greatly enable characterisation of complexes of molecular weight approaching *ca.* 1 MDa,^8^ greatly exceeding what is conventionally thought to be possible. There is therefore a need for straightforward synthetic methods to routinely prepare the necessary isotopically enriched precursors for such studies. Similarly, the emergence of deuterium-stabilised drug compounds^9^ and therapeutic peptides^10^ also places demand on new synthetic routes to isotopically labelled amino acids.

From a synthetic standpoint, chiral-deuteration of target precursors is challenging. Chemical strategies to prepare α-deuterated amino acids often rely on precious metals and expensive chiral ligands or auxillaries.^11–16^ Such approaches can suffer from imperfect selectivity, diminished isotopic purities, and complex work-up procedures, which can make the product cost prohibitively high for structural biology research. As an alternative to chemo-routes, several biocatalytic strategies have also been demonstrated for preparing α-deuterated amino acids.^17–21^ These methods benefit from the mild reaction conditions and inherent selectivity associated with enzyme reactivity, but the complex reaction mixtures hinder product isolation.

**Fig. 1:**
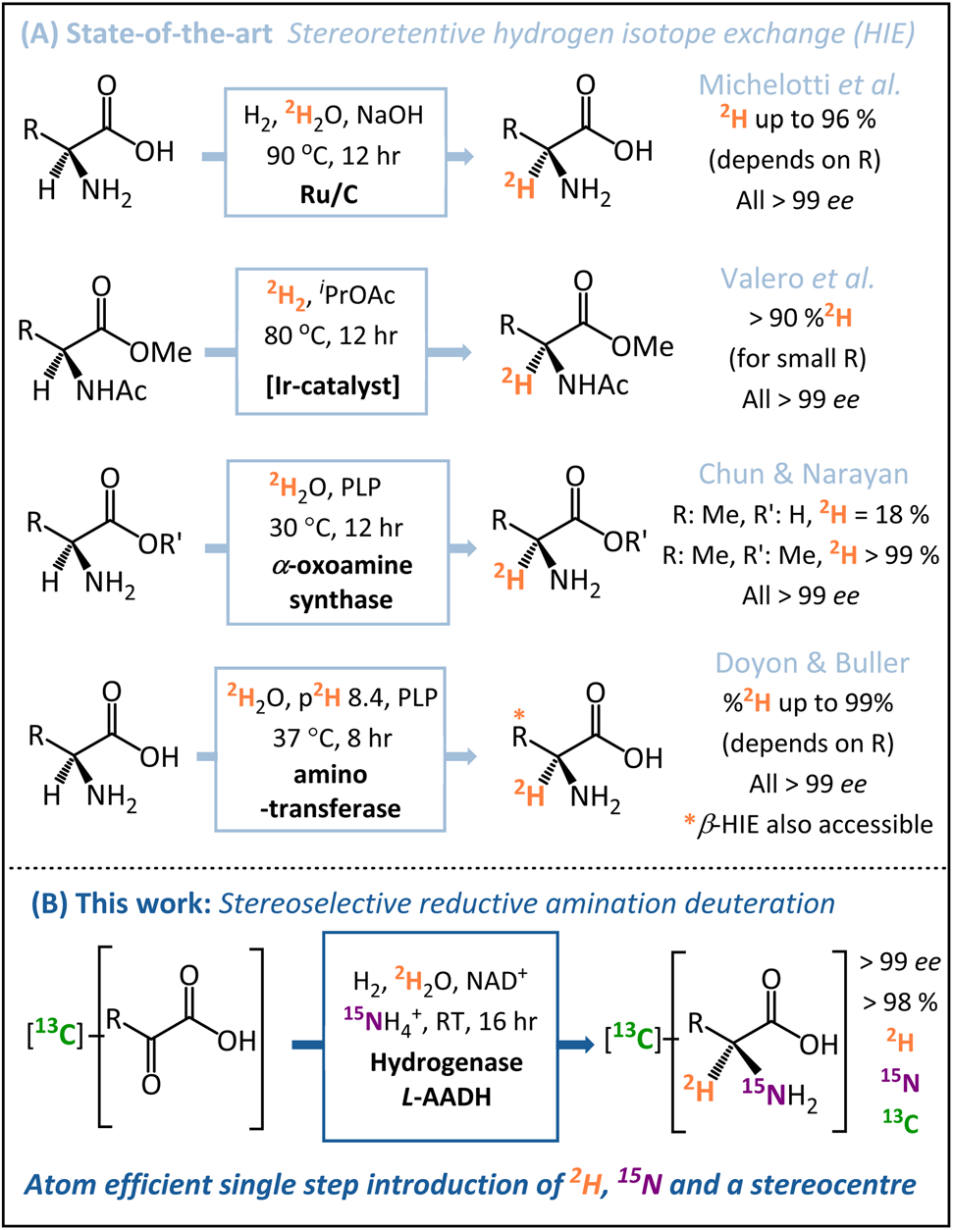
Routes to α-deuterated chiral amino acids (l-alanine in this case). (A) Stereoretentive hydrogen isotope exchange methodologies (HIE) utilise chemo- or bio-catalysts to install a ^2^H atom at a pre-formed asymmetric centre. (B) The stereoselective reductive amination approach used in this work installs the N-atom and ^2^H-atom with simultaneous formation of the chiral centre, enabling many labelling patterns from a generic starting compound.

A number of key advances have been made recently in hydrogen isotope exchange (HIE) strategies for the synthesis of isotopically labelled amino acids.^22–24^ Both Chun and Narayan^25^ and Doyon and Buller^26^ have published elegant biocatalytic HIE methods for stereoretentive α-deuteration of amino acids, both of which operate through pyridoxal phosphate (PLP)-dependent enzymes. The Doyon and Buller approach is particularly notable for the ability to control both α- and β-deuteration. Similarly, in the chemo-catalytic space, the groups of Roche^27^ and Pieters^14^ have shown ruthenium (supported on carbon or as nanoparticles on polyvinylpyrrolidone) to be particularly effective for the stereoretentive HIE of amino acids, and Valero *et al.* have demonstrated Kerr-type homogeneous iridium catalysts for similar reactions.^28^ These diverse state-of-the-art stereoretentive HIE strategies are summarised in Fig. 1A.

Whilst HIE strategies for the preparation of [^2^H]-amino acids have advanced significantly, such methods still require a pre-formed amine chiral centre prior to the installation of the deuterium. In the case of amino acids for protein NMR, multiple isotope labels {^2^H, ^15^N, ^13^C} are usually required,^29^ which are ideally installed in as few steps as possible from low-cost isotope pre-cursors. Motivated by this problem, we sought to develop a direct and inexpensive route to α-deuterated amino acids with high stereoselectively, minimal work-up requirements, and in-built versatility regarding the pattern of isotopic labels.

**Fig. 2:**
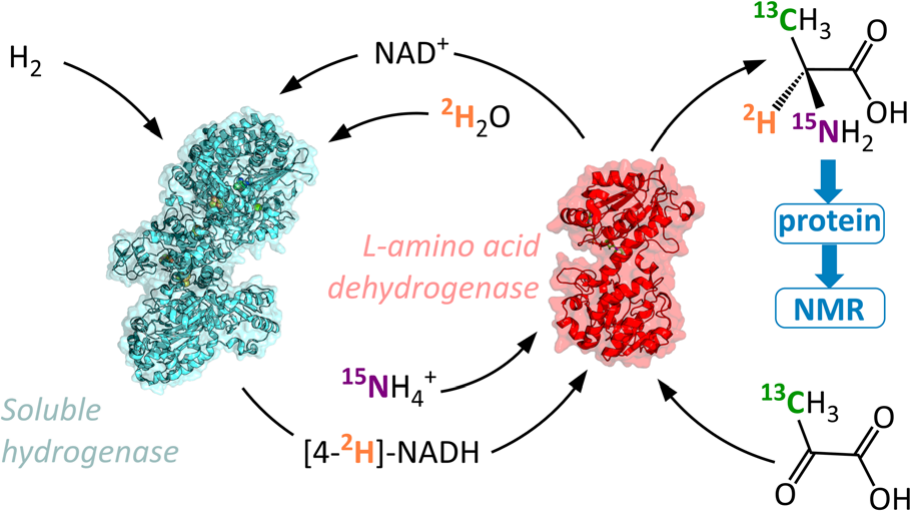
A H_2_-driven route to selectively isotopically labelled forms of l-alanine bearing an asymmetric deuterium centre. The scheme utilises H_2_ gas to drive the formation and recycling of [4-^2^H]-NADH from NAD^+^ and ^2^H_2_O by the action of a soluble hydrogenase.

In contrast to the HIE strategies employed elsewhere, we utilised a reductive amination approach to deuteration, whereby the N-atom, ^2^H-label, and chiral centre are all introduced into the target molecule in a single-step (Fig. 1B). This transformation is achievable by the use of an amino acid dehydrogenase (AADH); these are robust, widely available enzymes with naturally high activity and selectivity.^30–35^ The challenge in employing amino acid dehydrogenases for α-deuteration is the requirement to supply a suitably [^2^H]-labelled cofactor: [4-^2^H]-NADH. We have developed an atom-efficient strategy for generating [4-^2^H]-NADH from NAD^+^*in situ* using only H_2_ as a clean reductant and ^2^H_2_O as a cheap source of deuterium.^36^ The approach to obtain the deuterated cofactor uses an electronically coupled H_2_-oxidising site and NAD^+^ reducing site, in this case as part of the same enzyme, the soluble hydrogenase from *Ralstonia eutropha*, (now known as *Cupriavidus necator*), *Re*SH.^37,38^ The [4-^2^H]-NADH can then be supplied to an amino acid dehydrogenase (such as l-alanine dehydrogenase, l-AlaDH) in the presence of ^14^NH_4_^+^/^15^NH_4_^+^ and substrate to give a chiral [^2^H]-amino acid (Fig. 2).

This method effectively uses two enzymes in one solution to produce α-deuterated amino acids in gram quantities, with H_2_ in the local atmosphere providing the driving force. The approach is customisable, to allow for different ^2^H/^13^C/^15^N labelling patterns with little variation of the method. We demonstrate the effectiveness of this procedure by incorporating labelled l-alanine into a 400 kDa molecular chaperone, Hsp16.5, and acquiring a ^1^H–^13^C heteronuclear multiple quantum coherence (HMQC) spectrum suitable for high resolution structural and dynamical study. We anticipate that access to larger quantities of specifically labelled amino acids at substantially lower costs using our method will increase their use in biochemical studies.

## Results and discussion

### Verification of H_2_-driven biocatalytic system for producing a library of isotopically labelled amino acids

We have previously demonstrated that *Re*SH is capable of simultaneous reduction and isotopic labelling of NAD^+^ in ^2^H_2_O under low pressures of H_2_.^37,38^ Here, the action of the *Re*SH was coupled to that of an l-AlaDH in order to drive the reductive amination of pyruvate in the presence of ammonium bicarbonate (acting as both buffer and amine source). We selected l-alanine as the target compound, owing to the well-established strategies for up-take of this amino acid by *E. coli* and incorporation into proteins for NMR structure determination.^2^

**Fig. 3:**
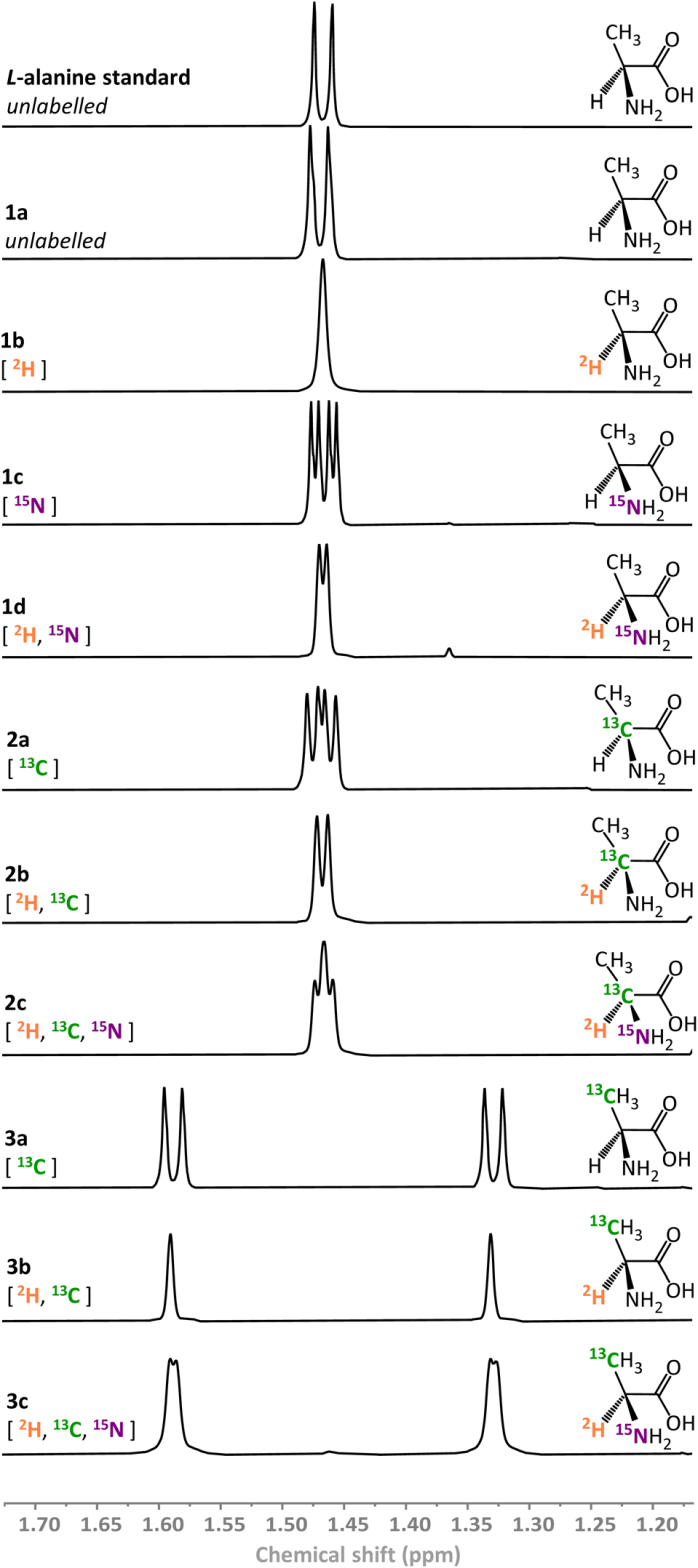
^1^H NMR spectra of variously isotopically labelled l-alanine molecules prepared biocatalytically (500 MHz, 293 K, ^2^H_2_O, p^2^H 8.0).

Using the combination of *Re*SH and l-AlaDH it was possible to generate a library of ten l-alanine isotopologues (compounds 1a–d, 2a–c, 3a–c in Fig. 3) simply by varying the solvent (H_2_O/^2^H_2_O), ammonium salt (^14^NH_4_^+^/^15^NH_4_^+^), and the pyruvate substrate (^12^C/^13^C). In each case, the ^1^H signals from the methyl peak of the alanine (–CH̲_3_, *δ* = 1.46 ppm) in the corresponding NMR spectra could be used to verify the product (full characterisation given in SI Section S.3.1†). Within the limits of detection, only a single isotopologue was formed in each case, verifying the high selectivity of the biocatalytic system, even in the presence of unlabelled H_2_ gas. It was found that the commercial l-AlaDH used to generate this library led to an enantiomeric excess (ee) of 90%, owing to the presence of a contaminating racemase. As such, a purified l-AlaDH (produced in house, see ESI Section S.2.2†) was used for all subsequent experiments, and delivered the expected >99% ee.

**Fig. 4:**
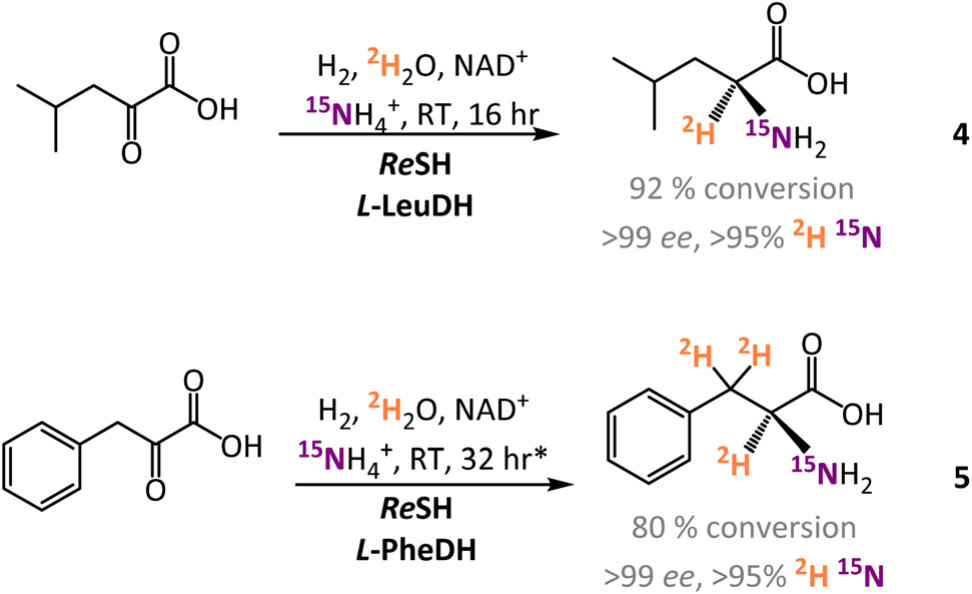
Preparation of ^2^H and ^15^N-labelled l-leucine and l-phenylalanine by H_2_ driven biocatalytic reductive amination and deuteration. *For 5, β-^2^H atoms were incorporated by pre-incubation of the substrate in ^2^H_2_O overnight before the enzymes were added.

In addition to the experiments with l-AlaDH, the H_2_-driven system was also coupled to commercial l-leucine dehydrogenase (l-LeuDH) and l-phenylalanine dehydrogenase (l-PheDH) enzymes. Using this system, it was straightforward to prepare l-[α-^2^H,^15^N]-leucine (4) and l-[α-^2^H,^15^N, β-^2^H_2_]-phenylalanine (5) with similarly high levels of conversion and isotopic and enantioselectivity (see Fig. 4 and ESI Section S.3.1†).

### Scale-up of H_2_-driven synthesis of α-deuterated amino acids

Following initial demonstration of the H_2_-driven biocatalytic system for preparing multiply labelled amino acid isotopomers, the reaction was tested on a preparative scale. Initially, 50 mg of 1d was prepared by reductive amination of pyruvate (25 mM) in ^2^H_2_O (25 mL, p^2^H 8.0) in a H_2_ atmosphere (2 bar) at room temperature, in the presence of [^15^N]H_4_HCO_3_ (50 mM), with *Re*SH, l-AlaDH, and NAD^+^ (0.1 mM). The role of the [^15^N]H_4_HCO_3_ was to serve as both an amine source and volatile buffer, enabling straightforward isolation of the product by rotary evaporation following the reaction. The reaction reached >99% conversion after 20 h, indicating a turnover number (ToN) for the *Re*SH of over 130 000.

This initial scaled-up reaction was set up anaerobically in a pressure-vessel, which may not be available in all laboratories. Therefore, in order to probe the wider applicability of the *Re*SH as a deuteration catalyst, a similar reaction to synthesise 3c was set up on the bench in standard laboratory glassware. Here, [3-^13^C]-pyruvate (35 mM) was reacted in a stirred solution of ^2^H_2_O (65 mL, p^2^H 8.0) in a round-bottom flask with a balloon of H_2_, under otherwise identical conditions. Again, this reaction proceeded to full conversion, and enabled the isolation of 198 mg of product.

Finally, the reaction conditions were further intensified to increase the amount of isolated product. Here, the synthesis of 1b was performed using 100 mM pyruvate in 60 mL ^2^H_2_O with 150 mM NH_4_HCO_3_, again in a round-bottom flask. Whilst full conversion was observed after 19 hours, giving rise to 520 mg of product (95% isolated yield), a slight decrease in ee was observed (97%) under the higher loading conditions. In all cases however, the isotopic purity of the isolated products (1d, 1b, 3c) closely followed the isotopic purity of the starting reaction mixture and simple precautions (such as washing the enzymes in ^2^H_2_O) were sufficient to keep the %^2^H high (>95%).

### Engineering isotopically enriched proteins with triply labelled l-alanine (3c) for methyl-TROSY

A specific application that stands to benefit tremendously from these methods is biomolecular NMR spectroscopy. Dipolar interactions between hydrogens dominate relaxation in proteins, where the slower they tumble, the broader the detected resonances in spectra. Typically, when a protein exceeds around 30 kDa, resonances are then unsuitable for study using conventional solution-state NMR methods.^39^ To overcome this limit, a combination of deuteration, and transverse relaxation optimised spectroscopy (TROSY) methods can be applied. Notably, the ‘methyl-TROSY’ methods developed by Kay *et al.*^40,41^ allow structural and dynamical characterisation of proteins approaching a molecular weight of 1 MDa,^8,42^ a limit which encompasses the majority of known proteins (excluding oligomers).^43^ This requires the selective incorporation of amino acids into proteins that contain ^13^C^1^H_3_ methyl groups, which are otherwise deuterated. Having isolated gram quantities of l-[α-^2^H,^15^N, β-^13^C]-alanine (3c), we sought to ascertain its suitability for solution-state methyl-TROSY experiments.

**Fig. 5:**
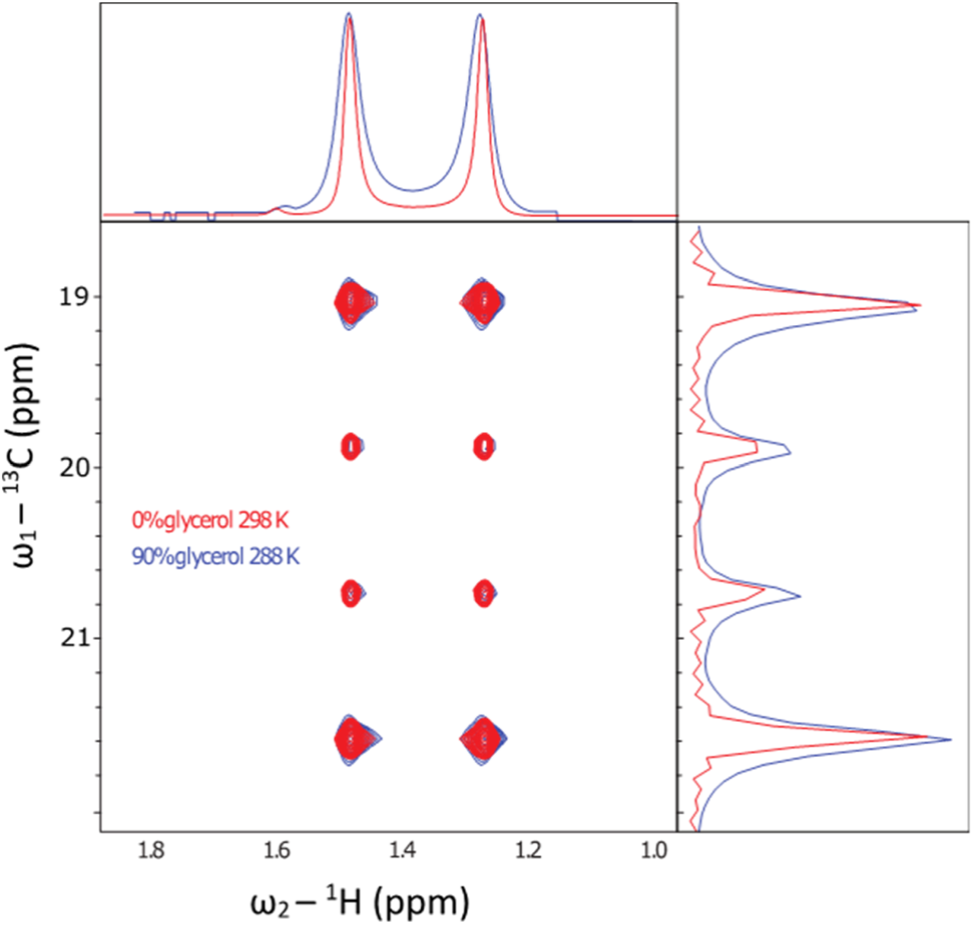
In order to simulate the rotational environment of the labelled amino acid inside a protein, l-[α-^2^H,^15^N, β-^13^C]-alanine was dissolved in a viscous glycerol medium and studied by NMR. The figure shows the ^13^C–^1^H decoupled HSQC experiment of the sample in ^2^H_2_O at 298 K (red) and 90 vol% glycerol at 288 K (blue trace). Under 0 vol% glycerol, the ratio of intensities is 3 : 1 : 1 : 3, as expected for a small molecule. At 90 vol% glycerol and low temperature, the resonances remained intense. The intensity ratio changed to approximately 2 : 1 : 1 : 2. Due to the outer resonances having 9× faster relaxation than the inner lines, in the macromolecular limit when the molecule tumbles slowly. Although the effects of slow tumbling are clear, the resonances remain sharp indicating the utility of this reagent inside high molecular weight proteins.

**Fig. 6:**
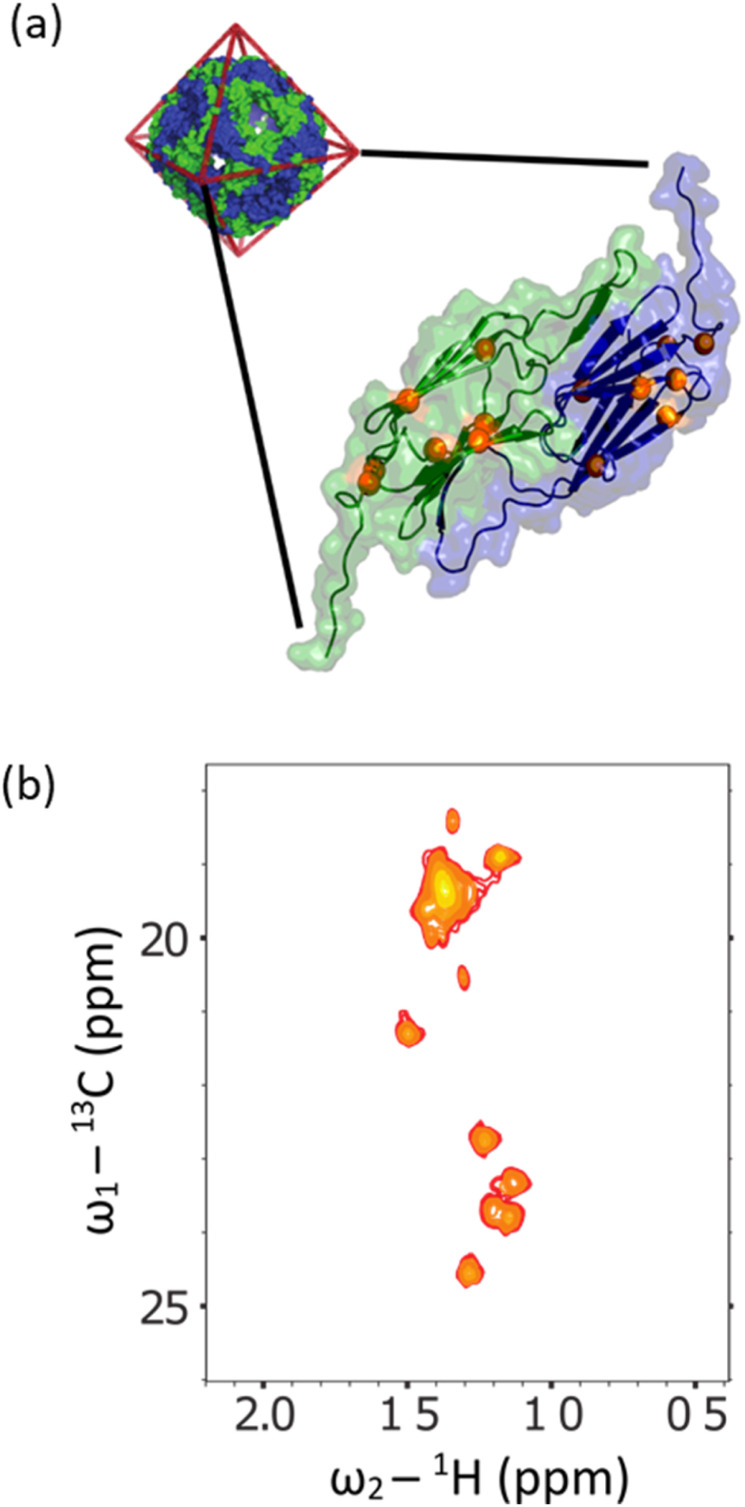
(a) The X-ray crystallographic structure of the small heat shock protein (HSP) from *M. jannaschii*, (PDB id 1SHS).^34^ The structure is built from dimeric sub-units, and the expected alanine ^13^CH_3_ groups are shown. The homo 24-mer has a molecular weight of 396 kDa. (b) Alanine region of methyl spectrum revealing 12 clear resonances with a range of linewidths. This contrasts with the X-ray structure that anticipates 8 unique environments. In solution, the complex adopts multiple conformations, and a range of environments with different motional regimes. This spectrum demonstrates that l-alanine produced using our synthesis scheme can be readily incorporated into high molecular weight biomolecules for mechanistic and structural studies.

Initially, a decoupled ^13^C–^1^H HSQC spectra of the methyl group of the sample were recorded at 298 K in ^2^H_2_O (Fig. 5, red trace), yielding a pair of quartets with signal intensity 3 : 1 : 1 : 3 in the ^13^C dimension, as expected for a small molecule where we detect an anti-phase coherence (which would be in the ratio 1 :3 : 3 : 1 in a ^13^C 1D spectrum detecting in-phase coherence). In a spectrum acquired at 288 K in 90 vol% glycerol (Fig. 5, blue trace), the spectra were highly similar. Under these conditions, the viscosity was increased by a factor of approximately 450, effectively simulating the local tumbling behaviour of the molecule as if it were part of a 50 kDa protein. This demonstrates that we expect sharp signals from this amino acid, even when incorporated in high molecular weight complexes. We therefore sought to incorporate this in a suitably chosen protein. We incorporated l-[α-^2^H,^15^N, β-^13^C]-alanine (3c) into the small heat shock protein Hsp16.5. This protein is a molecular chaperone from *Methanococcus jannaschii* that assembles into a 24-mer of molecular weight 396 kDa (Fig. 6a).^44^*In vitro*, this protein is a potent inhibitor of protein aggregation and amyloid formation. l-[α-^2^H,^15^N, β-^13^C]-Alanine (3c, 100 mg) was added 1 hour prior to induction, and the incorporation was >99%. A ^13^C–^1^H HMQC NMR spectrum was acquired. 12 resonances were clearly observed, with a range of linewidths, indicating differential mobility within different parts of the complex. As 8 unique resonances were expected from the symmetric structure characterised by X-ray crystallography, our precursor reveals multiple conformations of the protein (Fig. 6b). This spectrum validates the utility of this precursor in future biochemical studies. This is among the largest proteins for which an alanine methyl-TROSY spectrum has been recorded.^4,42^

## Conclusions and outlook

We have developed a convenient means for the production of selectively deuterated amino acids. We demonstrated the success of the method in preparing variously ^2^H, ^15^N, and ^13^C-labelled l-alanines, and further showed that the principle behind the technique is applicable to a wider range of amino acids. Our scheme exploits enzymes that can be produced with simple molecular biology strategies in most laboratories, uses easily implemented reaction conditions and workup procedures, and cheap and readily available isotopic precursors. The technique installs the ^2^H and ^15^N labels simultaneously with the formation of the chiral centre, and enables wide versatility in the choice of isotopes added. We anticipate this approach facilitating rapid access to arrays of variously labelled canonical and non-canonical amino acids. This in turn will enable mechanistic, structural, and biophysical studies of large biomolecules such as proteins and peptides in biologically relevant environments.

## Data availability

The datasets supporting this article have been uploaded as part of the ESI.†

## Author contributions

J. S. R., K. A. V., H. A. R., and A. J. B. jointly conceptualised and supervised the work. J. S. R., J. H. N., K. U., and P. M. T. T. synthesised amino acids. M.A.R. performed molecular biology. G. K. and A. J. B. carried out protein NMR. All authors contributed to data analysis and assisted with the drafting of the manuscript.

## Conflicts of interest

A patent application by J. S. R., H. A. R. and K. A. V. detailing some of this research was filed through Oxford University Innovation (Feb 2018). The remaining authors declare no competing interests.

## Supplementary Material

SC-014-D3SC01718D-s001
