# Supplementary material for: Biocatalytic reductive amination as a route to isotopically labelled amino acids suitable for analysis of large proteins by NMR
Source: Chem Sci. 2023 Oct 26;14(43):12160–5. doi: 10.1039/d3sc01718d (PMC10631221; doi:10.1039/d3sc01718d)
Supplement: SC-014-D3SC01718D-s001 [file SC-014-D3SC01718D-s001.pdf]

Electronic Supplementary Material (ESI) for Chemical Science.
This journal is © The Royal Society of Chemistry 2023

Supplementary Information (SI)

Biocatalytic reductive amination as a route to isotopically labelled amino acids suitable for analysis of large proteins by NMR

Jack S. Rowbotham,* Jake H. Nicholson, Miguel A. Ramirez, Kouji Urata, Peter M.T. Todd, Gogulan Karunanithy, Lars Lauterbach, Holly A. Reeve, Andrew J. Baldwin,* Kylie A. Vincent*

*Correspondence to: jack.rowbotham@manchester.ac.uk, andrew.baldwin@chem.ox.ac.uk kylie.vincent@chem.ox.ac.uk

Contents

S.1 Abbreviations **2**

S.2 Materials and Methods **2**

S.2.1 General solvents and reagents 2
S.2.2 Preparation of enzymes for biocatalytic reactions 2
S.2.2.1 Amino acid dehydrogenases 2
S.2.2.2 Soluble hydrogenase from Ralstonia eutropha, ReSH 2
S.2.3 Biocatalysis reaction conditions 3
S.2.4 Analysis of products from biocatalysis 4
S.2.5 Expression and purification of sHsp16.5 4
S.2.6 NMR analysis of sHSP16.5 5

S.3 Supplementary data **6**

S.3.1 Characterisation of isotopically labelled amino acids 6
S.3.1.1 Characterisation of isotopic alanine library 6
S.3.1.2 Characterisation of isotopically labelled *L*-leucine and *L*-phenylalanine 10
S.3.1.3 Characterisation of isolated *L*-[α-^{2}H]-alanine samples (1b, 1d, 3c) 14

S.4 Supplementary references **16**

S.1 Abbreviations

**NAD^+**	Nicotinamide adenine dinucleotide (oxidised form)
**NADH**	Nicotinamide adenine dinucleotide (reduced form)
**[4-^{2}H]-NADH**	Nicotinamide adenine dinucleotide (reduced form) deuterated in the 4 position of the nicotinamide ring
***Re*SH**	Soluble hydrogenase from *Ralstonia eutropha*
***L*-AlaDH**	*L*-alanine dehydrogenase
***L*-PheDH**	*L*-phenylalanine dehydrogenase
***L*-LeuDH**	*L*-leucine dehydrogenase
**HSP 16.5**	Small heat shock protein from *methanococcus jannaschii*
E. coli	*Escherichia coli*
R. eutropha	*Ralstonia eutropha*
**TROSY**	Transverse relaxation-optimized spectroscopy
**HMQC**	Heteronuclear Multiple Quantum Coherence
**RPM**	revolutions per minute

S.2 Materials and Methods

S.2.1 General solvents and reagents

General reagents, buffer salts, and isotopically labelled pre-cursors were purchased from Sigma Aldrich and NAD^+ was purchased from Prozomix, and all were used as received without further purification. All non-deuterated solutions were prepared with MilliQ water (Millipore, 18 MΩcm), and deuterated solutions with ^2H_2O (99.98 %, Sigma Aldrich). All solvents were deoxygenated by sparging with dry N_2 for 60 minutes prior to use. Buffer solutions of $[^{15}N]H_4HCO_3$ (50 mM, p^{2}H 8.0) in ^2H_2O were prepared from $[^{15}N]H_4Cl$ by exchanging the Cl^- with an anion exchange resin (Dowex 1×8, HCO_3^- form). All other reagents and solvents were purchased from Sigma Aldrich and used as received, unless specified otherwise.

S.2.2 Preparation of enzymes for biocatalytic reactions

S.2.2.1 Amino acid dehydrogenases

Commercial *L*-alanine dehydrogenase (Sigma Aldrich, recombinant from *E. coli*) and *L*-leucine and *L*-phenylalanine dehydrogenases (Johnson Matthey) were obtained in their lyophilised form and used as received. The recombinant expression of the NADH-dependent alanine dehydrogenase from *Bacillus subtilis* (Uniprot KB Q08352) was carried out in *E. coli*, according to previously reported methods.[1]

S.2.2.2 Soluble hydrogenase from Ralstonia eutropha, ReSH

For the production of *Ralstonia eutropha* H16 Soluble Hydrogenase (*Re*SH), megaplasmid-free strain *R. eutropha* HF210 carrying an overexpression plasmid for strep-tagged SH (*R. eutropha* HF904) was inoculated from glycerol stocks into three 2.8 L flasks each containing 1.5 L of FGN media (63 mM Na_2HPO_4, 11 mM KH_2PO_4 at pH 7.0 supplemented with 0.05%

w/v fructose, 0.4% v/v glycerol, 0.2% w/v NH_4Cl, 0.8 mM $MgSO_4$, 68 µM $CaCl_2$, 40 µM $FeCl_3$, 1 µM $NiCl_2$, 1 µM $ZnCl_2$, and the trace element solution[2] supplemented with tetracycline (10 µg mL^{-1}). After inoculation, flasks where incubated in a shaker at 180 rpm, 30°C until the optical density at 436 nm (OD_{436}) reached approximately 10 absorbance units (8 days of incubation). Subsequently, cells were harvested by centrifugation at 6000 × g for 30 minutes and stored at -80°C.

Frozen cells were thawed on ice and re-suspended in lysis buffer (50 mM Tris-HCl at pH 8.0, 150 mM KCl, 5% glycerol). Cells were disrupted by two cycles of sonication (Fisherbrand™ Q500 Sonicator fitted with standard 0.5-inch probe; Amp 30, 2-second pulse, 5-second pause, total sonication of 5 minutes). Cellular debris were collected by centrifugation at 18000 × g for 1 hour at 4°C. The soluble lysate was filtered through a 0.45 *µ*m porous membrane and applied to a previously equilibrated 10 mL Strep-Tactin Superflow column (IBA). The bound protein was washed with 5 column-volumes of lysis buffer. Pure SH protein was eluted using lysis buffer containing 2.5mM desthiobiotin (IBA). Subsequently, the pure protein was concentrated and buffer-exchanged into lysis buffer with Ultra Centrifugal Filter Units (EMD Millipore Amicon™, 100 kDa exclusion size). Pure enzyme was flash-frozen in liquid nitrogen and stored at -80°C.

Whilst the soluble hydrogenase from *ReSH* is not currently commercially available, several alternatives may be purchased, including (i) a heterogeneous H_2-driven cofactor recycling system available from HydRegen Ltd. (which we have previously reported on),[3,4] and (ii) a NAD^+-reducing soluble hydrogenase from *pyrococcus furiosis*, currently available from Kerafast Inc.

Reasonable requests for materials (catalysts or deuterated compounds) can also be made through the corresponding authors.

S.2.3 Biocatalysis reaction conditions

Screening reactions were set up in a glovebox under a protective N_2 atmosphere (O_2 < 0.1 ppm) and were conducted on a 1000 µL scale in sealed 1.5 mL micro-centrifuge tubes (Eppendorf) punctured with five holes in the lid (Ø 1.0 mm). In a typical procedure, reaction solutions containing NH_4HCO_3 (50 mM, pH 8.0) were pre-saturated with H_2 gas, and NAD^+ (0.1 mM) and pyruvic acid (20 mM) were added. Suitable isotopically labelled precursors/buffers combinations were used depending on the required product. The *Re*SH was added at a loading of 16 µg mL^{-1} and the *L*-AlaDH was added at a loading of 50 - 100 µg mL^{-1}. The punctured tubes were then transferred to a H_2-atmosphere (2 bar) within a pressure vessel (Tinyclave steel, Büchi AG) and rocked back and forth at 45 rpm whilst the reactions took place (16 hours, 21 °C). Reactions were adapted to prepare ^{2}H and ^{15}N-labelled *L*-leucine (**4**) and *L*-phenylalanine (**5**) by using 4-methyl-2-oxovaleric acid and phenylpyruvic acid as substrates and *L*-LeuDH and *L*-PheDH as enzymes, respectively. In the case of **5**, the phenylpyruvic acid was incubated in the reaction mixture for 16 hours prior to adding the enzymes in order to enable ^{2}H incorporation by keto-enol tautomerisation. Reactions were carried out on a 1 mL scale with 10 mM of substrate and 20 mM of NH_4HCO_3.

Selected *L*-alanine isotopologues were also prepared at scale and isolated, by adapting the same protocols accordingly:

- **1d** was prepared by reductive amination of a solution of pyruvic acid (25 mM, 25 mL, 0.63 mmol) in ^2H_2O at p^{2}H 8.0 under a H_2 atmosphere (2 bar) at 21 °C, in the presence of [^{15}N]H_4HCO_3 (50 mM), with *Re*SH, *L*-AlaDH, and NAD^+ (0.1 mM). The reaction was set-up anaerobically in a glovebox, and was conducted in the glass liner of a pressure vessel with constant stirring (400 rpm). After 20 hours the reaction reached full conversion (by ^{1}H NMR), and the [^{15}N]H_4HCO_3 buffer solution was removed by repeated co-evaporation with H_2O at 60 °C. The crude off-white product was washed with acetone and dried in air at 60 °C, leaving behind **1d** (55 mg, 0.60 mmol) as a white solid in a 97% yield.

- **3c** was prepared by performing an aerobic reaction in standard laboratory glassware. Here, a solution of [3-^{13}C]-pyruvic acid (35 mM, 65 mL, 2.28 mmol) in ^2H_2O buffered to p^{2}H 8.0 was reacted in a round bottom flask with a balloon of H_2, and the above biocatalysts at the same concentration. The reaction proceeded to full conversion over 20 hours, and a similar workup enabled the isolation of **3c** (198 mg, 2.15 mmol) in a 94% yield.
- Finally, **1b** was synthesised using similar conditions to **3c** with a more concentrated solution of pyruvic acid (100 mM, 60 mL, 6.0 mmol) in ^2H_2O at p^{2}H 8.0, containing NH_4HCO_3 (150 mM) in a round bottom flask. Full conversion was observed by ^{1}H NMR after 19 hours, and a similar work-up gave rise to 520 mg **1b** product as a 95 % isolated yield.

The isolated samples of **1d**, **3c**, and **1b** were subsequently analysed by mass spectrometry, NMR spectroscopy, and chiral GC (following the procedures in Section S.2.4) to confirm their identity and purity.

S.2.4 Analysis of products from biocatalysis

Products from small-scale demonstration reactions were characterised without isolation of the product. Firstly, samples were subjected to analysis by ^{1}H NMR and, where possible, ^{2}H and ^{13}C NMR on either a Bruker Avance III HD nanobay (400 MHz) or Bruker Avance III (500 MHz) NMR spectrometer, utilising previously reported parameters.[3]

For GC-MS and chiral-GC-FID analysis, samples were first derivatised with ethyl chloroformate/ethanol according to the method of Bertand *et al*[5] to give the N-ethoxycarbonyl ethylester. Here, an aliquot of reaction mixture (100 µL) was mixed with EtOH (23 µL) and pyridine (13 µL) by means of a vortex mixer. Ethyl chloroformate (15 µL) was added, and the reaction was allowed to stand at room temperature until bubbles stopped forming (around 30 mins). The derivatised product was then extracted with 100 *µ*L of CDCl3, and transferred to a glass vial for analysis by GC. Chiral GC-FID was carried out on a ThermoScientific Trace 1310 fitted with a CP-Chirasil-Dex CB column (Agilent), 25 m length, 0.25 mm diameter, 0.25 *µ*m (film thickness) and a guard of 10 m deactivated fused silica of the same diameter. GC-MS was carried out on an Agilent 7890B GC coupled to an Agilent 7200 Accurate Mass Q-ToF MS operating under EI mode, and fitted with a DB-1701 column (Agilent), 30 m length, 0.25 mm diameter, 0.25 µm (film thickness). The full parameters for the requisite GC methods have been reported previously.[3]

In the case of ^{2}H and ^{15}N-labelled *L*-leucine (**4**) and *L*-phenylalanine (**5**), the reactions were analysed in the same way as the the *L*-alanine samples described above. The only exception was the Chiral-GC analysis of *L*-phenylalanine. Here, the derivatisation procedure was carried out using methyl chloroformate/methanol and the samples were analysed on an Agilent 6850 GC (with autosampler) using the following conditions: Injector: 200 °C, 13 psi He, 50:1 split, 2 – 5 µL injection; Column: BetaDEX^{TM} 325 (Supelco) 30 × 0.25 mm × 0.25 *µ*m; Carrier gas: He 13 psi (1.2 mL/min); Oven: 5 min at 70 °C, ramp to 180 °C at 1 °C/min, hold 25 min; Detection: FID.

Isotopically labelled samples of alanine isolated from scale-up reactions were analysed by ^{13}C and ^{1}H NMR after redissolving them in ^2H_2O. The same samples were studied by HRMS on a Thermo Exactive Mass Spectrometer, calibrated to a mass accuracy of ≤ 5 ppm. Finally, the *ee* of the samples were verified by chiral-GC-FID after deriviatisation by ethyl chloroformate/ethanol as described above.

S.2.5 Expression and purification of sHsp16.5

Plasmid pET-Hsp16.5 encoding the small heat-shock protein (sHSP) from *Methanococcus jannaschii* was transformed into competent BL21(DE3) *E. coli* cells (Agilent Technologies LDA UK Limited) and plated on LB-agar solid media supplemented with ampicillin (100 *µ*g mL^{-1}; Fisher Scientific UK Ltd). After overnight incubation at 37°C, a single colony was inoculated

into 50 mL of LB media supplemented with ampicillin (100 μg mL^{-1}) and further incubated at 37°C, shaking at 220 RPM. Once the optical absorbance at 600nm (OD_{600}) reached 0.5, 10 mL of this culture were collected by centrifugation (2000 × g, 10 minutes) and re-suspended with 50 mL of M9 minimal media previously equilibrated at 37°C and supplemented with ampicillin (100 µg mL^{-1}). The culture was incubated at 37°C, 220 RPM and grown to OD_{600} 0.5. Doubling times for this culture were approximately 1 hour. Once the culture reached OD_{600} 0.5, 20 mL were collected by centrifugation (2000 × g, 10 minutes) and re-suspended with 100 mL of deuterated M9 media (^2H_2O -M9) previously warmed to 37°C, and supplemented with ampicillin (100 µg mL^{-1}) and trace LB powder (25 mg L^{-1}). ^2H_2O-M9 media was prepared by mixing the solid ingredients directly into ^2H_2O followed by filter-sterilisation. The ^2H_2O-M9-based bacterial culture was incubated at 37°C, 220 RPM overnight. The following morning, the bacterial culture OD_{600} was ~3.3, indicating successful assimilation of the bacteria to deuterium-based media. A fraction of the overnight culture (approximately 33 mL) was centrifuged and re-suspended with 1L of ^2H_2O-^{13}C-M9 media (containing 0.3% w/v ^{13}C glucose), previously warmed to 37°C and supplemented with ampicillin (100 µg mL^{-1}) and trace LB powder (25 mg L^{-1}). This culture was further incubated at 37°C, 220 RPM. Once the OD_{600} reached 1.0 (approximately eight hours of incubation), the labelled alanine (100 μg mL^{-1}) and isoleucine precursor (50 μg mL^{-1}) were added to the culture. One hour later, isopropyl-β-d-thiogalactopyranoside (IPTG) was added for protein induction (0.24 g L^{-1}). The culture was further incubated at 37°C, 220 RPM for 13 hours. After incubation, the final OD_{600} was ~2.8. At this point, cells were harvested by centrifugation at 6000 × g for 30 minutes and stored at -80°C.

The bacterial pellet was re-suspended in 20mM Tris-HCl (pH 8.0) buffer and lysed by two cycles of sonication. The cell debris were collected by centrifugation at 18000 × g for 30 minutes at 4°C. The soluble lysate was filtered through a 0.45 μm porous membrane and applied to a previously equilibrated 5-mL HiTrap Q HP Anion Exchange chromatography column using an ÄKTA Start protein purification system (GE Healthcare Life Sciences). Once the soluble lysate was loaded, the column was washed with 50 mL of 20 mM Tris-HCl buffer (pH 8.0) followed by a linear gradient from 0 mM NaCl to 300 mM NaCl buffer containing 20mM Tris-HCl (pH 8.0). The target protein was eluted isocratically at 300 mM NaCl, 20mM Tris-HCl (pH 8.0). Fractions containing the target protein were pooled and concentrated to 1 mL volume using Ultra Centrifugal Filter Units (100 kDa exclusion size), and further enriched by size exclusion chromatography in a HiLoad 16/600 Superdex 200PG column (GE Healthcare Life Sciences). Fractions that eluted at the expected molecular mass (~400 kDa) were collected from a single peak, and buffer-exchanged into NMR Sample Buffer (50 mM Sodium Phosphate, 2mM EDTA, 2mM NaN_3 in ^2H_2O) using Ultra Centrifugal Filter Units.

S.2.6 NMR analysis of sHSP16.5

All NMR spectroscopy experiments were recorded on a 14.1 T Varian Inova spectrometer. Decoupled ^{13}C HSQC spectra (Fig 5) and ^{13}C HMQC (Fig 6) spectra were processed with NMRPipe and visualised with Sparky.

S.3 Supplementary data

S.3.1 Characterisation of isotopically labelled amino acids

S.3.1.1 Characterisation of isotopic alanine library

Table S.1 Summary of NMR signals observed for isotopic alanine library

	**δ ^{1}H (ppm) 500.13 MHz**		**δ ^{2}H (ppm) 76.77 MHz**	**δ ^{13}C (ppm) 125.76 MHz**		
	methyl hydrogens	α-hydrogen	α-hydrogen	C1 (COOH)	C2 (α)	C3 (Me)
**1a**	1.46 (*d*, *J* = 7.2)	3.76 (q, *J* = 7.3)	n.o.	175.83	50.66	16.28
**1b**	1.46 (s)	n.o.	3.62 (*s*)	175.87	50.36 (*t*, *J* = 22.3)	16.15
**1c**	1.46 (*dd*, *J* = 7.3, 3.1)	3.75 (*q*, *J* = 7.2)	n.o.	176.17	50.74 (*d*, *J* = 5.4)	16.44
**1d**	1.46 (*d*, *J* = 3.0)	n.o.	3.74 (*s*)	175.99	50.50 (*td*, *J* = 22.1, 5.7)	16.39
**2a**	1.45 (*dd*, *J* = 7.6, 4.3)	3.75 (*dq*, *J* = 145, 7.2)	n.o.	n.o.	50.90	n.o.
**2b**	1.45 (*d*, *J* = 4.4)	n.o.	3.62 (*d*, *J* = 22.6)	n.o.	50.19 (*t*, *J* = 22.2)	n.o.
**2c**	1.45 (*dd*, *J* = 4.4, 3.1)	n.o.	3.63 (*d*, *J* = 22.8)	n.o.	50.17 (*td*, *J* = 22.6, 5.9)	n.o.
**3a**	1.45 (*dd*, *J* = 129.6, 7.2)	3.75 (*m*)	n.o.	n.o.	n.o.	16.42
**3b**	1.46 (*d*, *J* = 129.6)	n.o.	3.63 (*s*)	n.o.	n.o.	16.07
**3c**	1.46 (*dd*, *J* = 129.7, 2.9)	n.o.	3.63 (*s*)	n.o.	n.o.	16.04

***J* = coupling constant (in Hz), *s* = singlet, *d* = doublet, *t* = triplet, *q* = quartet, *m* = multiplet, n.o. = not observed**

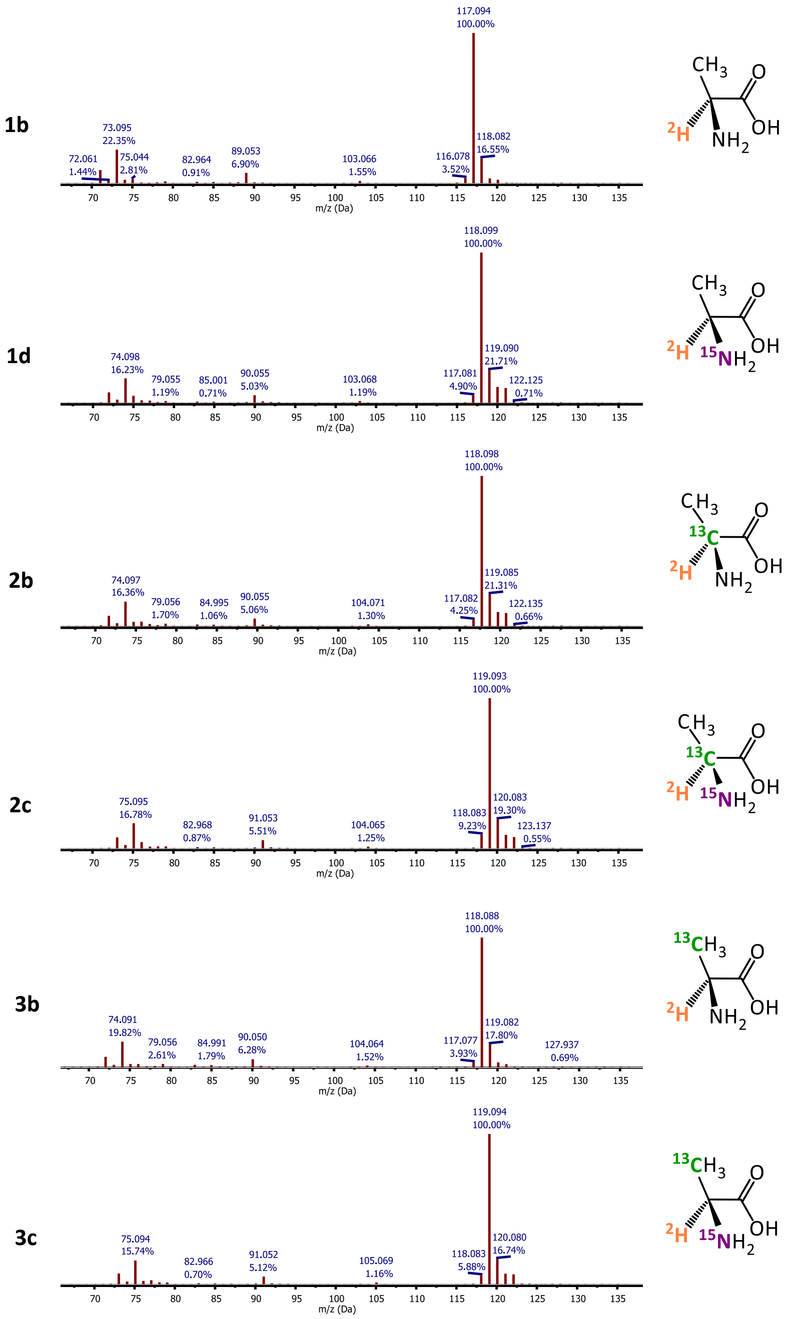

Figure S. 1 MS analysis of *α*-deuterated *L*-alanine samples (following derivatisation with ethanol/ethyl chloroformate). For reference, natural abundance *L*-alanine derivatised by the same method gives a peak with m/z = 116.08, corresponding to [$C_5H_{10}NO_2^{+\bullet}$].[3]

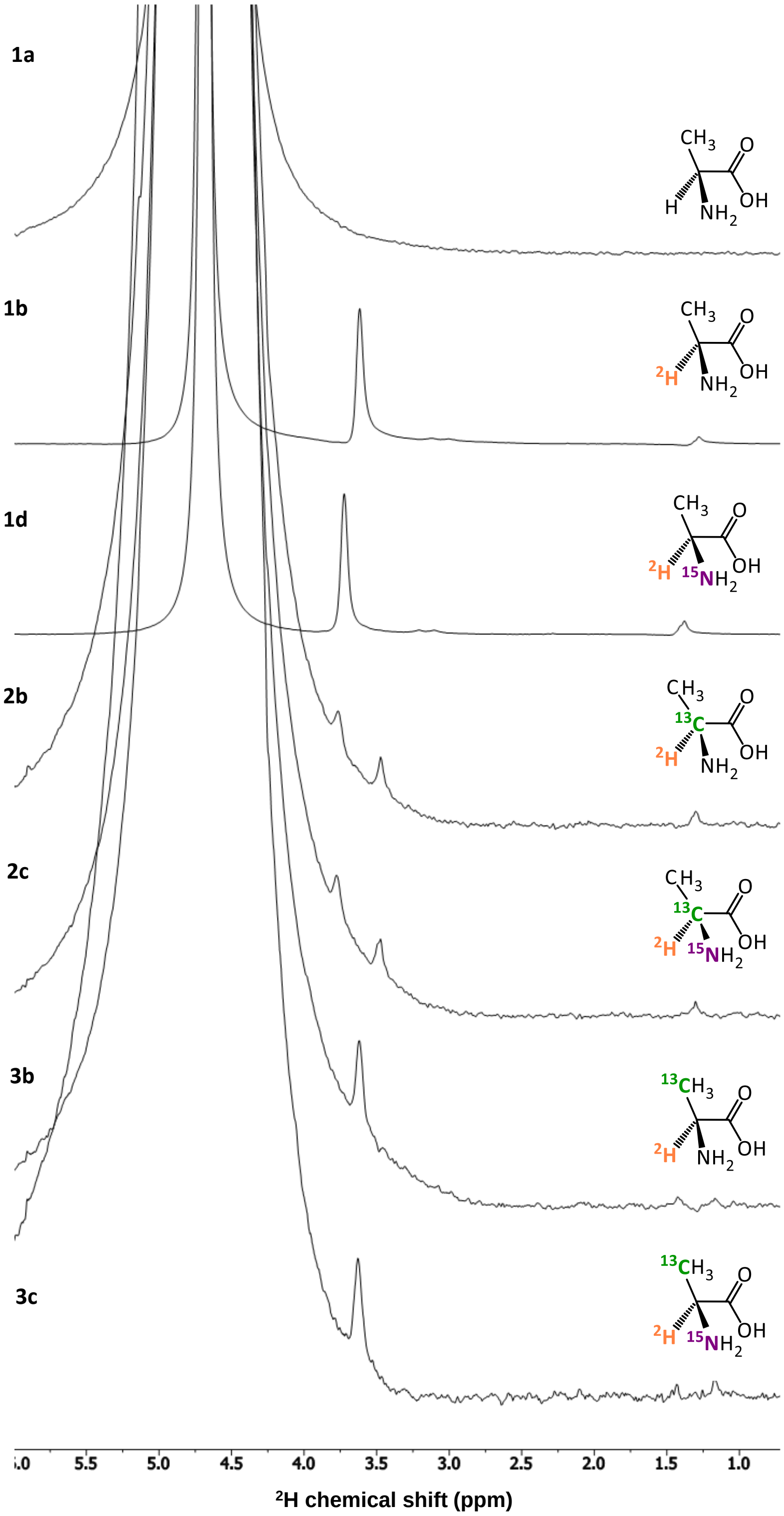

Figure S. 2 ^{2}H NMR analysis (H_2O/^2H_2O, p^{2}H 8.0, 76.75 MHz, 293 K) of *α*-deuterated *L*-alanines produced by the biocatalytic system. **2b**, **2c**, **3b**, and **3c** were dissolved in neat ^2H_2O, all others were dissolved in H_2O: ^2H_2O (90:10 v/v)

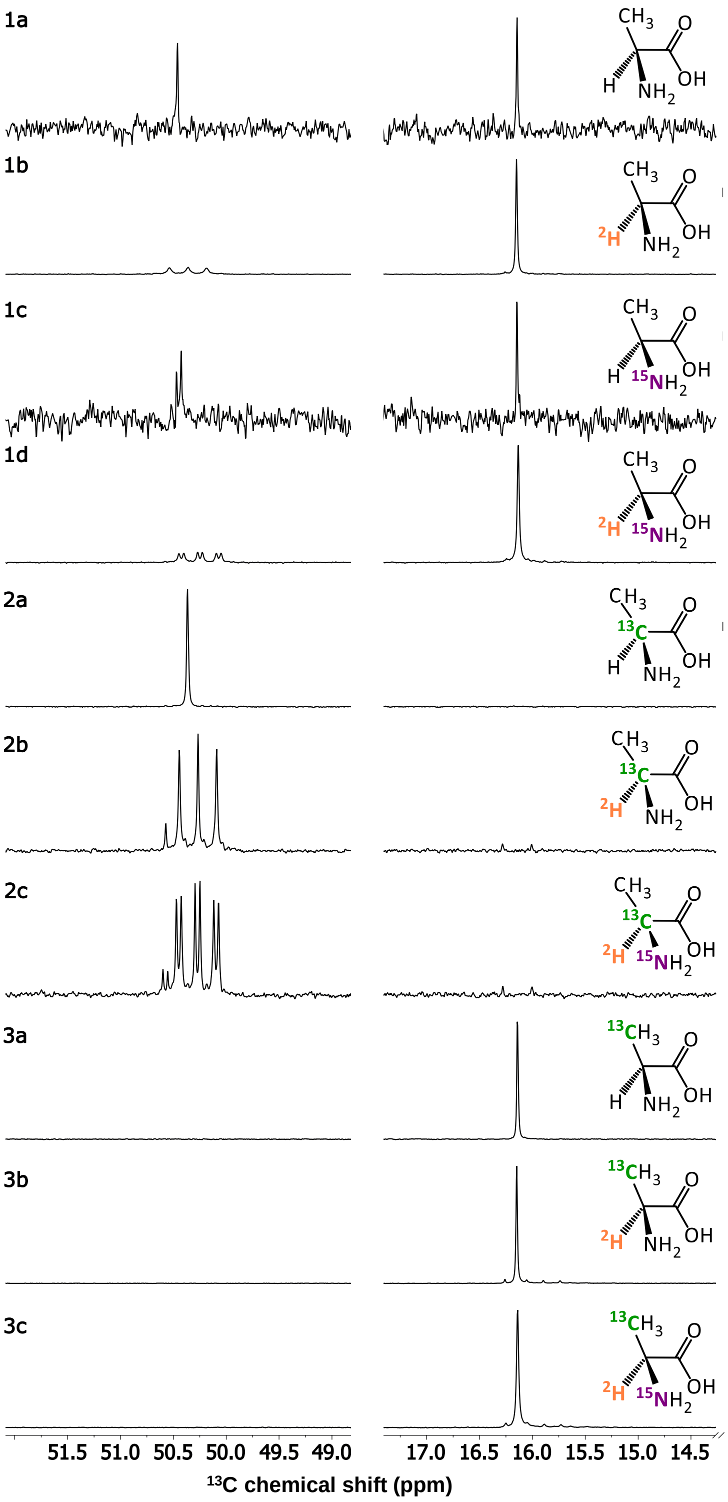

Figure S. 3 ^{13}C NMR analysis (H_2O/^2H_2O, p^{2}H 8.0, 125.72 MHz, 293 K) of *L*-alanines produced by the biocatalytic system. **2b**, **2c**, **3b**, and **3c** were dissolved in neat ^2H_2O, all others were dissolved in H_2O: ^2H_2O (90:10 v/v).

S.3.1.2 Characterisation of isotopically labelled *L*-leucine and *L*-phenylalanine

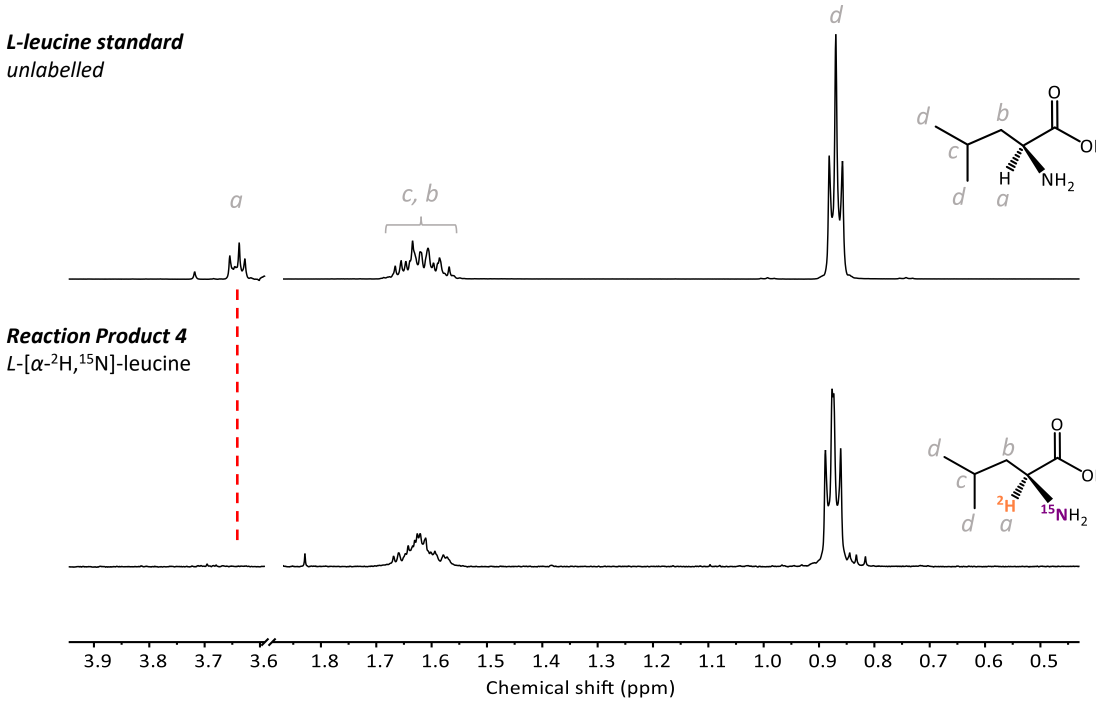

Figure S. 4 ^{1}H NMR analysis (^2H_2O, p^{2}H 8.0, 400 MHz, 293 K) of reaction mixture to prepare *L*-[*α*-^{2}H,^{15}N]-leucine (**4**). The absence of the signal at 3.65 ppm in the bottom (reaction) spectrum relative to the top (*L*-leucine standard) spectrum is diagnostic of the deuteration of the *α*-carbon in the product.

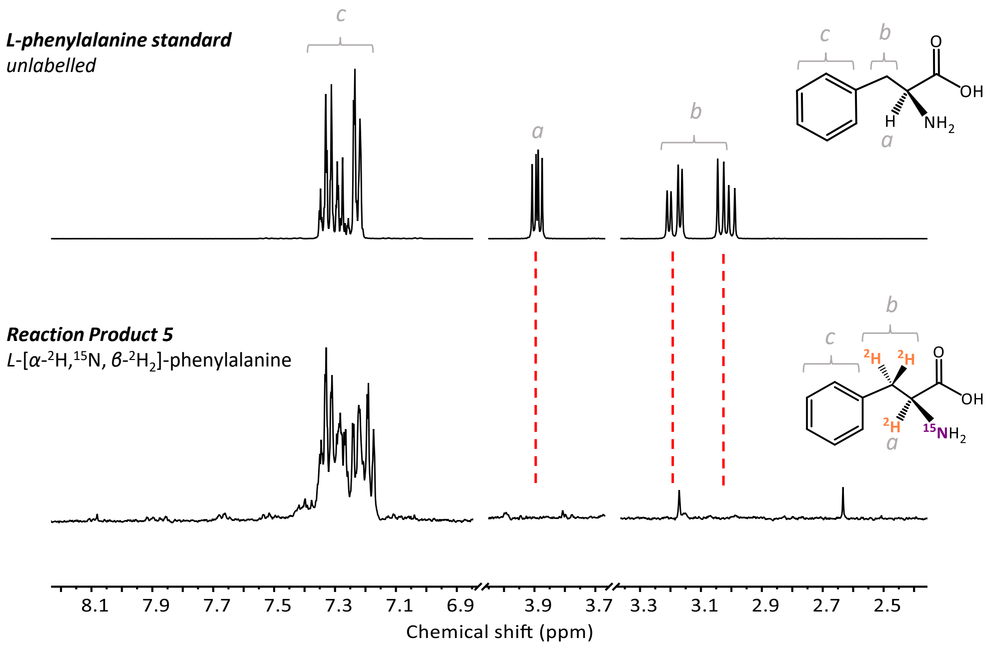

Figure S. 5 ^{1}H NMR analysis (^2H_2O, p^{2}H 8.0, 400 MHz, 293 K) of reaction mixture to prepare *L*-[*α*-^{2}H,^{15}N, *β*-$^2H_2$]-phenylalanine (**5**). The absence of the signals at 3.00, 3.20, and 3.90 ppm in the bottom (reaction) spectrum relative to the top (*L*-phenylalalnine standard) spectrum is diagnostic of the deuteration of the *α*- and *β*-carbons in the product.

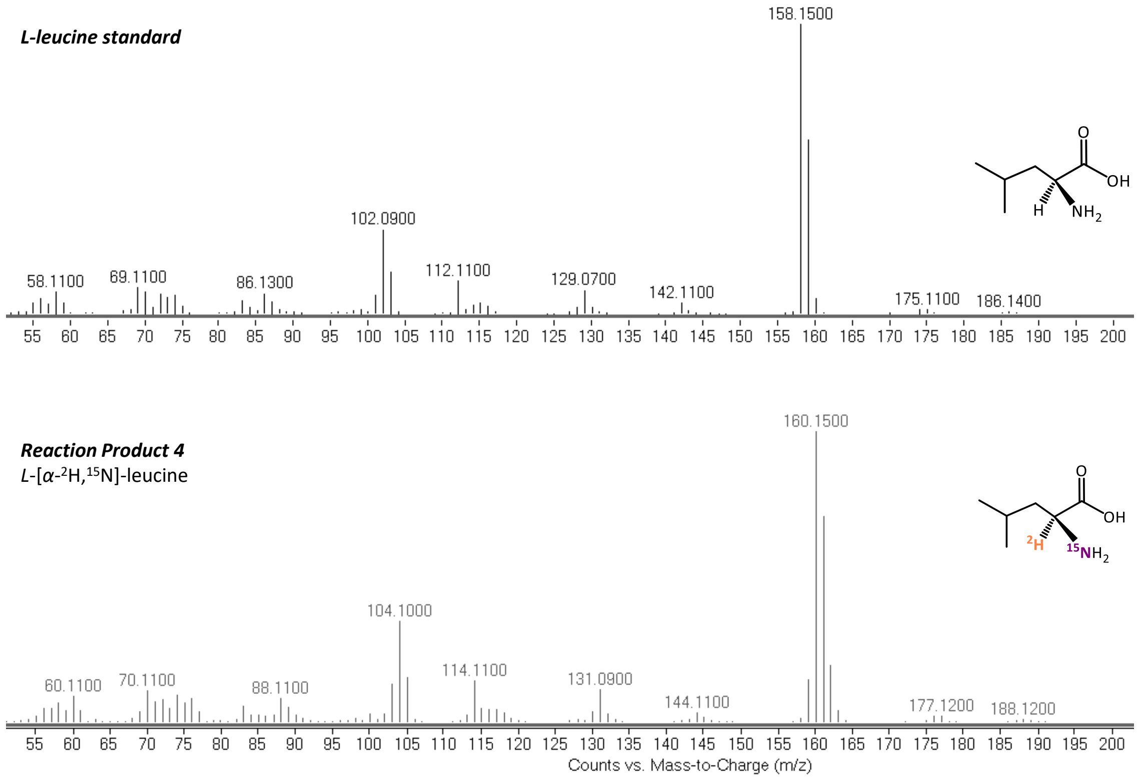

Figure S. 6 MS analysis reaction mixture to prepare *L*-[*α*-^{2}H,^{15}N]-leucine (**4**) (following derivatisation with ethanol/ethyl chloroformate).The +2 mass shift for the signal at m/z 160.15 in the bottom trace (reaction) relative to the top trace (*L*-leucine standard) is diagnostic of the incorporation of ^{2}H and ^{15}N into the product.

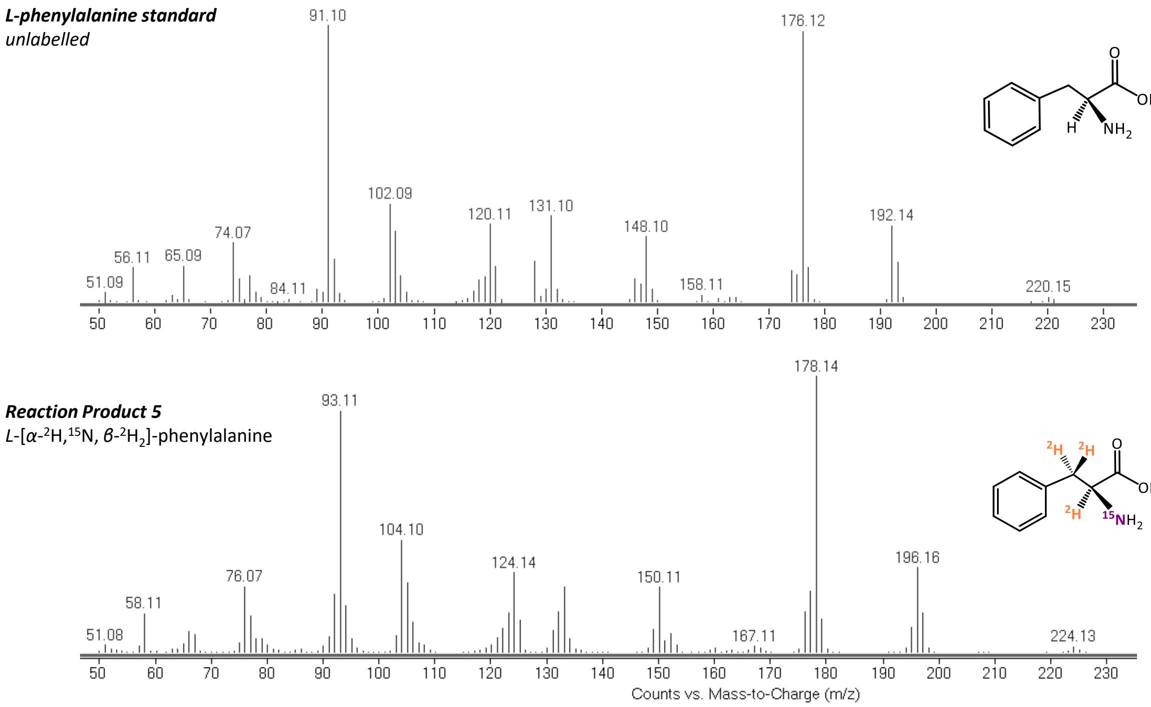

Figure S. 7 MS analysis of reaction mixture to prepare *L*-[*α*-^{2}H,^{15}N, *β*-$^{2}H_2$]-phenylalanine (**5**) (following derivatisation with ethanol/ethyl chloroformate).The +4 mass shift for the signal at m/z 196.16 in the bottom trace (reaction) relative to the top trace (*L*-phenylalanine standard) is diagnostic of the incorporation of three ^{2}H and one ^{15}N atoms into the product.

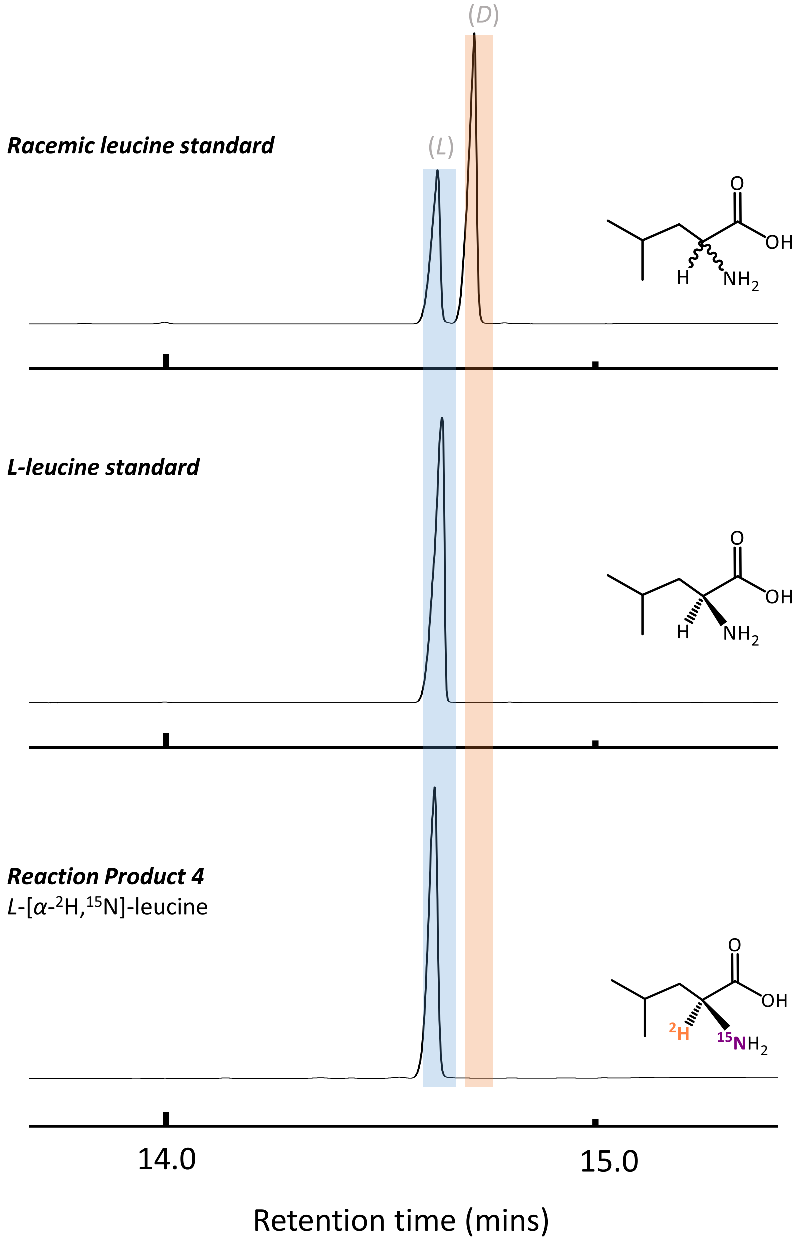

Figure S. 8 Chiral GC analysis of reaction mixture to prepare *L*-[*α*-^{2}H,^{15}N]-leucine (**4**) (following derivatisation with ethanol/ethyl chloroformate). The bottom chromatogram (reaction mixture) demonstrates that the reaction proceeds with full selectivity for the *L*-isomer.

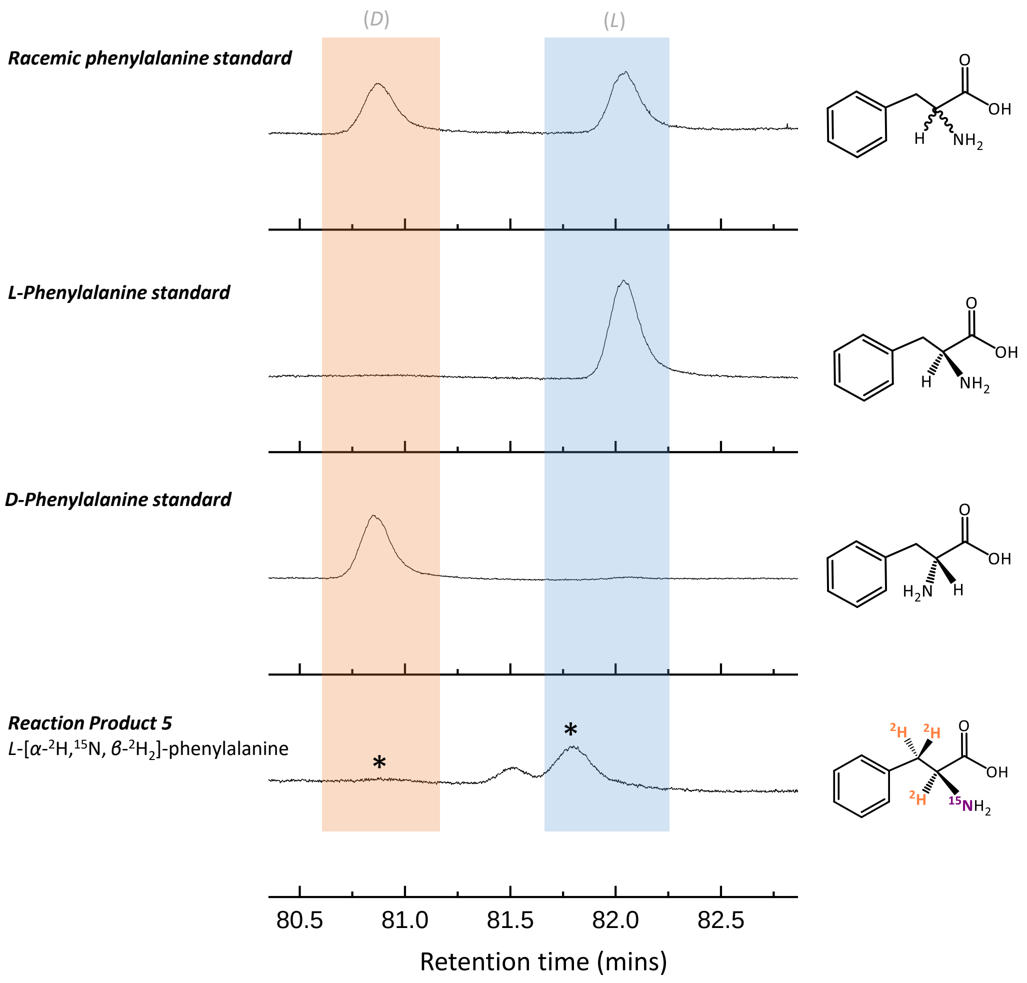

Figure S. 9 Chiral GC analysis of reaction mixture to prepare *L*-[α-^{2}H,^{15}N, β-^{2}H$_2$]-phenylalanine (**5**) (following derivatisation with methanol/methyl chloroformate). The bottom chromatogram (reaction mixture) demonstrates that the reaction proceeds with full selectivity for the *L*-isomer. *The assignment of these peaks was further verified by doping in small quantities of *D*- and *L*-isomer.

S.3.1.3 Characterisation of isolated *L*-[*α*-^{2}H]-alanine samples (1b, 1d, 3c)

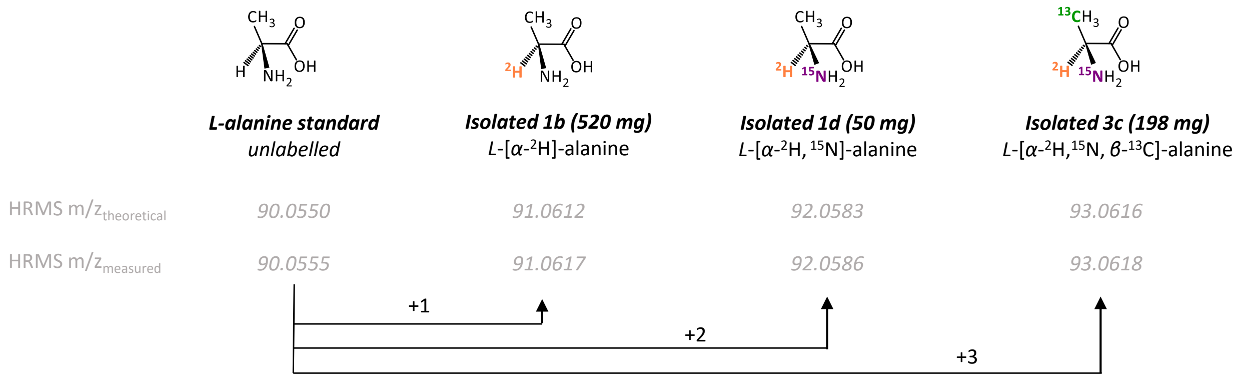

Figure S. 10 Summary of *L*-[*α*-^{2}H]-alanine isotopologues isolated following H_2-driven biocatalysis reactions. Analysis by HRMS shows the expected +1 (**1b**), +2 (**1d**) and +3 (**3c**) mass shifts for the reaction products relative to an unlabeled commercial sample of *L*-alanine.

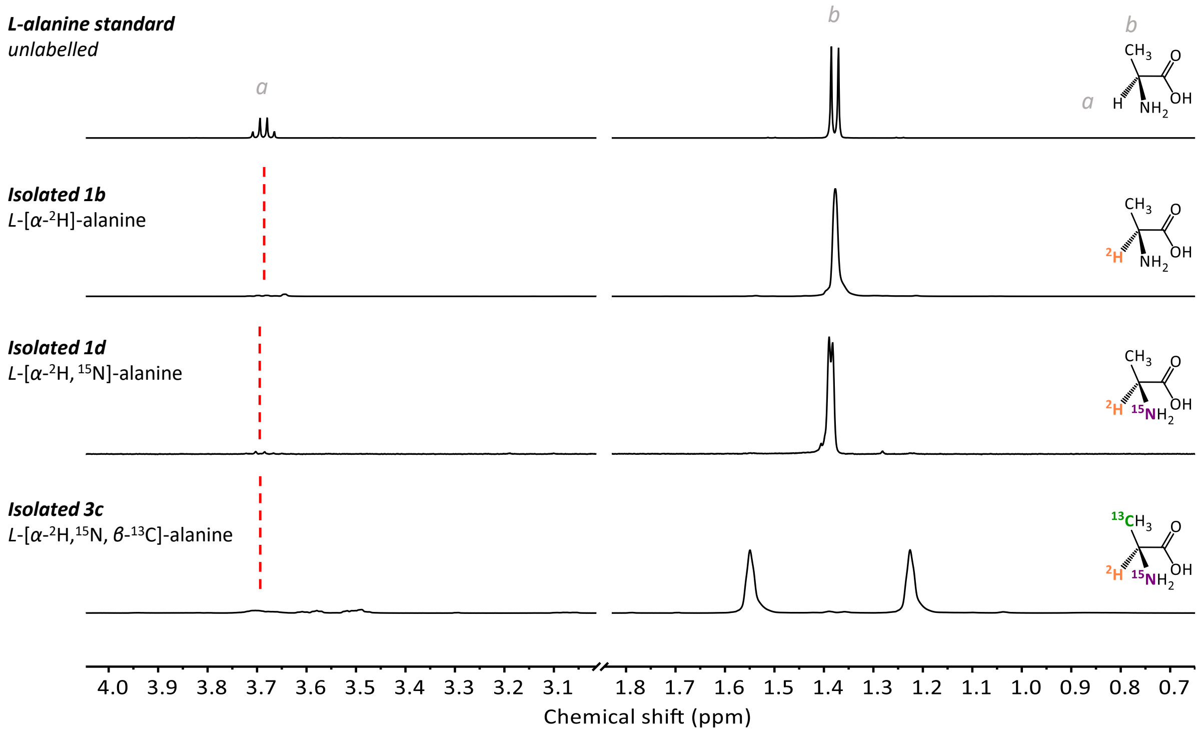

Figure S. 11 ^{1}H NMR analysis ($^{2}H_2O$, 400 MHz, 293 K) of isolated *α*-deuterated *L*-alanines produced by the biocatalytic system (**1b**, **1d**, and **3c**) compared to unlabeled commercial *L*-alanine standard. The spectra are consistent with the analysis in Table S.1.

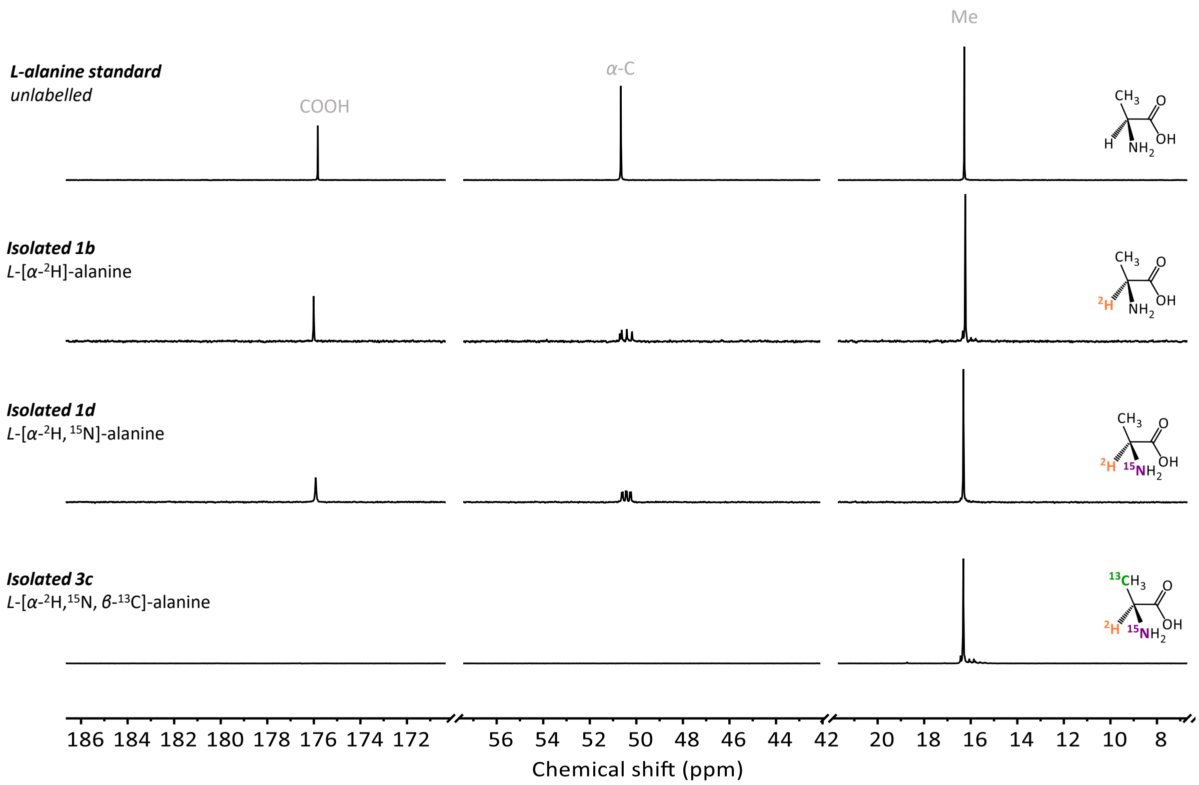

Figure S. 12 ^{13}C NMR analysis (^2H_2O, 100 MHz, 293 K) of isolated *α*-deuterated *L*-alanines produced by the H_2-driven biocatalytic system (**1b**, **1d**, and **3c**) compared to unlabeled commercial *L*-alanine standard. The spectra are consistent with the analysis in Table S.1.

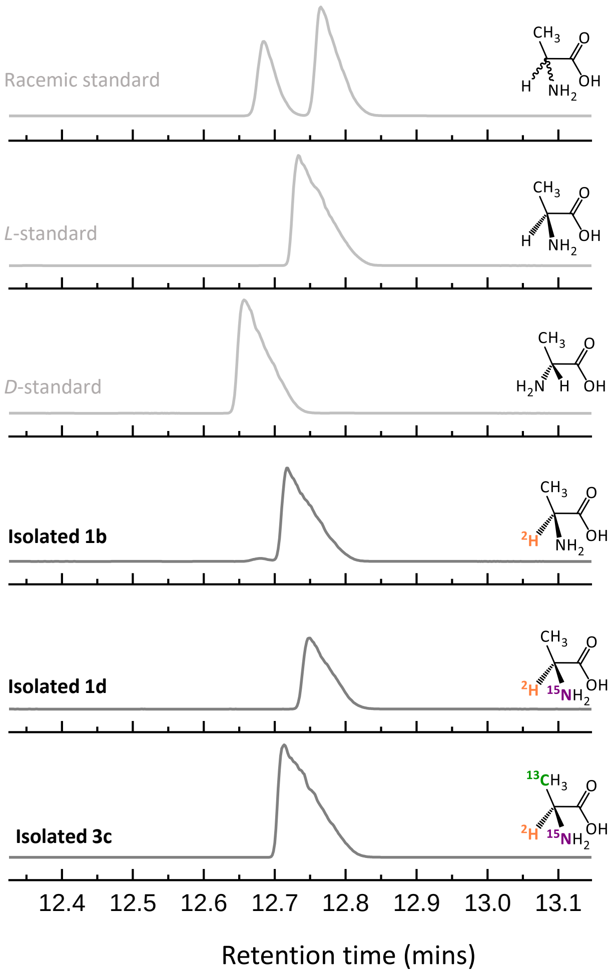

Figure S. 13 Chiral GC analysis of isolated *L*-[*α*-^{2}H]-alanine samples **1b**, **1d**, and **3c** (following derivatisation with ethanol/ethyl chloroformate). The lower three chromatograms demonstrate the stereopurity of the *L*-isomer products (**1b** *ee* = 97; **1d** *ee* = 100; **3c** *ee* = 100).

S.4 Supplementary references

1. Thompson, L. A. *et al.* Rapid, Heterogeneous Biocatalytic Hydrogenation and Deuteration in a Continuous Flow Reactor. *ChemCatChem* **12**, 3913–3918 (2020).
2. Pfennig, N. Rhodopseudomonas globiformis, sp. n., a New Species of the Rhodospirillaceae. *Arch. Microbiol.* **100**, 197–206 (1974).
3. Rowbotham, J. S., Ramirez, M. A., Lenz, O., Reeve, H. A. & Vincent, K. A. Bringing biocatalytic deuteration into the toolbox of asymmetric isotopic labelling techniques. *Nat. Commun.* **11**, 1454 (2020).
4. Rowbotham, J. S., Reeve, H. A. & Vincent, K. A. Hybrid Chemo-, Bio-, and Electrocatalysis for Atom-Efficient Deuteration of Cofactors in Heavy Water. *ACS Catal.* **11**, 2596–2604 (2021).
5. Bertrand, M., Chabin, A., Brack, A. & Westall, F. Separation of amino acid enantiomers VIA chiral derivatization and non-chiral gas chromatography. *J. Chromatogr. A* **1180**, 131–137 (2008).
